# A Systematic Approach for the Interpretation of Cardiopulmonary Exercise Testing in Children with Focus on Cardiovascular Diseases

**DOI:** 10.3390/jcdd10040178

**Published:** 2023-04-19

**Authors:** Bibhuti B. Das

**Affiliations:** Division of Pediatric Cardiology, Department of Pediatrics, McLane Children’s Baylor Scott and White Medical Center, Baylor College of Medicine-Temple, Temple, TX 76502, USA; bibhuti.das@bswhealth.org; Tel.: +1-254-935-4980; Fax: +1-254-935-4941

**Keywords:** exercise testing, exercise physiology, cardiopulmonary exercise testing, children

## Abstract

Cardiopulmonary exercise testing (CPET) is the clinical standard for children with congenital heart disease (CHD), heart failure (HF) being assessed for transplantation candidacy, and subjects with unexplained dyspnea on exertion. Heart, lung, skeletal muscle, peripheral vasculature, and cellular metabolism impairment frequently lead to circulatory, ventilatory, and gas exchange abnormalities during exercise. An integrated analysis of the multi-system response to exercise can be beneficial for differential diagnosis of exercise intolerance. The CPET combines standard graded cardiovascular stress testing with simultaneous ventilatory respired gas analysis. This review addresses the interpretation and clinical significance of CPET results with specific reference to cardiovascular diseases. The diagnostic values of commonly obtained CPET variables are discussed using an easy-to-use algorithm for physicians and trained nonphysician personnel in clinical practice.

## 1. Introduction

The Exercise Is Medicine (EIM) logo is published by the American College of Sports Medicine (ACSM) to emphasize the importance of remaining active and being happy [1]. The roots of exercise physiology, dating back thousands of years to the ancient Indus River civilization, are well documented in the literature. Susruta, a surgeon from India (approximately 600 BC), prescribed moderate exercise to establish an equilibrium between three humors: Bayu (air), Pitta (bile), and Kapha (phlegm) [2]. The three humoral theory was later endorsed by the father of scientific medicine, the famous Greek physician of the fifth century BC, Hippocrates [3]. The religion of Taoism from China (1050-256 BC) also believed exercise helped to eliminate “bad air” [4]. Claudius Galenus, a Greek physician and philosopher (AD131-201), described that arteries carry oxygen, which is essential for healthy body functioning [5]. The intriguing feature of history is how the physiologic effects of exercise have been regarded and accepted to promote health. The nineteenth century has been described as the beginning of scientific medicine, followed by a sufficient number of investigations worldwide, indicating that exercise is an integral part of human well-being. However, due to several factors, lack of exercise is more prevalent in today’s society than ever in human history. The *World Obesity Atlas 2022*, published by the World Obesity Federation, predicts that one billion people globally, including 1 in 5 women and 1 in 7 men, will be living with obesity by 2030 [6]. In the US, between 2017–2020, the prevalence of obesity was 19.7% and affected about 14.7 million children and adolescents aged 2–19 years [7]. There are numerous global organizations, including the World Health Organization (WHO), National Institute of Health (NIH), US Department of Human Services, ACSM, American Association of Cardiovascular and Pulmonary Rehabilitation (AACVPR), American College of Cardiology (ACC), American Heart Association (AHA), American Academy of Pediatrics (AAP), European Society of Cardiology (ESC), Exercise and Sports Science of Australia (ESSA), Canadian Cardiovascular Society (CCVS), Canadian Society for Exercise Physiology (CSEP) and many others that have identified the significant role of exercise as a treatment option for the prevention of many diseases as well as improved health care outcomes. Many cardiovascular risks factors, such as atherosclerotic lesions in the aorta and coronary artery vessels, begin early in life, as evidenced by the Bogalusa Heart Study [8]. The Coronary Artery Risk Development in Young Adults study showed that cardiovascular (CV) disease risk increases significantly in the presence of any of these six risk factors: hypertension, hyperlipidemia, smoking, being overweight, having a sedentary lifestyle, and an unhealthy diet [9]. As CV diseases increase worldwide and one-third of the world’s population remains physically inactive [2], screening exercise testing programs for the early identification of at-risk populations in childhood is the first step towards improving health and quality of life.

This review paper describes a systematic approach to interpreting cardiopulmonary exercise testing (CPET) in children. The CPET combines standard graded CV stress testing to assess a patient’s ability to tolerate increased intensities of aerobic exercise, including monitoring ECGs for ischemia/arrhythmia and hemodynamic monitoring (blood pressure, thus systemic vascular resistance) with simultaneous ventilatory respired gas analysis. This review does not describe the details of pretest procedures, indications, contraindications, and risks of exercise testing. Readers are referred to the AHA guidelines (discussed later) for further information on standard CV exercise testing in children. Because heart, lung, skeletal muscle, peripheral vasculature, and cellular metabolism impairment frequently lead to circulatory, ventilatory, and gas exchange abnormalities during exercise, an integrated analysis of the multi-system response to exercise can be beneficial for differential diagnosis of exercise intolerance.

## 2. Physiological Basis of CPET

Cardiorespiratory physiology varies from resting to exercising in an adolescent patient (Table 1). How these changes are achieved constitutes the physiology of exercise. Table 1 describes the differences in cardiopulmonary variables between resting and exercise.

The human body behaves under physical stress as an integrated system that delivers oxygen (O_2_) to mitochondria to perform aerobic exercise [10]. The O_2_ supply to the exercising muscles depends on blood flow, hemoglobin concentration, partial pressure of O_2_ in the patient’s arterial blood, and the adequate release of O_2_ to working muscles. During CPET, breath-by-breath, the VO_2_ and the volumes of carbon dioxide (CO_2_) production and air expired (minute ventilation (VE)) are estimated systematically. This integrative approach and analysis of the different physiological systems are more valuable than evaluating each physiological system separately at rest. The noninvasive and dynamic nature of the performed measurements provides the clinician with important information that can be used for diagnostic, prognostic, and evaluative purposes. Subsequently, CPET may reveal alterations in multiple body functions, including the cardiovascular, respiratory, muscular, metabolic, and neuro-hormonal systems [11,12]. As a result, exercise tolerance is determined by three factors: pulmonary gas exchange, circulation of blood, and O_2_ extraction by skeletal muscle.

Cellular respiration gradually breaks down a glucose molecule into CO_2_ and water. Along the way, ATP is produced by oxidative phosphorylation (aerobic) vs. substrate-level phosphorylation (anaerobic). The anaerobic energy pathways have a much higher power (rate of ATP production) but a smaller capacity (total ATP produced) than the aerobic pathways [13]. The continual supply of ATP to the fundamental cellular processes underpins skeletal muscle contraction during exercise. The movement of electrons powers ATP production through the electron transport chain, a series of proteins embedded in the inner membrane of the mitochondrion. The intensity and duration of exercise primarily determine the relative contribution of these metabolic pathways. In healthy individuals, ATP resynthesis is closely matched to the ATP demand of exercise. At the anaerobic threshold (AT), as the O_2_ demand of the exercising muscle exceeds the O_2_ supply, anaerobic metabolism is used to supply the energy required to continue. The lactic acid produced is mainly buffered, predominantly by the bicarbonate system, resulting in a rise in CO_2_ in the capillary and venous blood. The compensatory ventilatory mechanisms try to maintain homeostasis of CO_2_ by increasing ventilation. AS exercise progresses, further the lactic acid gradually increases. The respiratory system responds to two CO_2_ sources: (1) the metabolic CO_2_ generated from aerobic metabolism and (2) the excess CO_2_ resulting from buffered lactic acid. This is the basis of determining an individual’s fitness to sustain higher fractional VO_2_ for a longer time without reaching the AT and can have high endurance before tiredness or fatigue.

## 3. Clinical Guidelines for Exercise Testing in Children

Clinical exercise testing is a relatively new field and continues to evolve. However, several guidelines published in the literature from scientific organizations help implement exercise testing in children as young as six. In 2006, the AHA Council on cardiovascular disease in the young committee on Atherosclerosis, Hypertension, and Obesity in Youth recommended clinical training and competency for physicians, setting laboratories for exercise testing, equipment, pretest procedures, laboratory staffing, indications, and contraindications, relative risks of stress testing, and stress protocols [14]. That being said, the 2006 AHA guidelines need to be updated with new findings currently available from research in exercise physiology. Therefore, there is general recognition that the relentless progress of exercise testing leads to the irrelevance of clinical practice guidelines that only undergo periodic review and updating.

Several CPET protocols exist, and many exercise laboratories use standardized tests. When the child’s performance is compared with reference values, it is necessary to standardize the CPET protocol to match the testing procedures and methodology used to establish the reference values [15,16,17,18,19]. For comparison, there are also normal predicted values for each CPET variable [20]. It is also essential to select the appropriate CPET protocol to evaluate a child’s complaints and symptoms while considering their physical fitness. For example, although the Bruce protocol is the most frequently used treadmill protocol for a CPET in children and adolescents [21] for differential diagnostics in pediatrics, a cycle ergometer is preferred. The cycle ergometer possesses multiple clinical advantages over treadmill testing in pediatric clinical settings, including the fact that the test will not be constrained by mechanical limitations of a patient (e.g., inefficient gait; deformities; soreness in ankles, knees, and hip, or balance problems); the risk for injuries is considered negligible; the peak work rate (WRpeak) can be obtained precisely; and it is easier to obtain better quality physiological measurements, including electrocardiography and blood pressure (e.g., fewer movement artifacts). However, it is worth mentioning that the cycle ergometer test does result in lower PVO_2_ than treadmill running in children.

Furthermore, the speed of the treadmill protocol is often a restrictive factor for young children, next to the need for familiarity. When performing a CPET using a cycle ergometer, the Godfrey protocol [22] is sometimes used in children and adolescents. In the ramp modification, there is a minor increase in the WR in shorter intervals instead of increases per minute; this protocol is more compatible with modern electronically braked cycle ergometers equipped with automated protocols. The ramp modification allows a more precise examination of the patient’s exercise response, especially in those with severe limitations and/or deconditioning. In addition to an electronically braked cycle ergometer, the CPET equipment should include a metabolic cart, which is able to analyze respired gases (O_2_ and CO_2_) with a rapid response time to provide breath-by-breath measurements of ventilatory gas exchange variables, as well as ancillary equipment for serial monitoring of the electrocardiogram, blood pressure, and peripherally measured oxygen saturation (SpO_2_) [14,23].

## 4. Parameters Obtained by Cardiopulmonary Exercise Tests

Table 2 summarizes the CPET-derived variables obtained during a symptom-limited maximal CPET. (Note: the reference values for the CPET parameters used in the following discussion are mainly derived from adults and applied in analyzing CPET in my practice. Children need to have more pediatric reference values independent of their body size and pubertal stage. Furthermore, there needs to be more validation from different protocols used in children. Therefore, each laboratory should use its standard reference values and z-score equations for the CPET parameters and consistently interpret them.)

Among these variables, the basic parameters, which are mandatory for interpretations, are peak oxygen consumption (PVO_2_), the volume of CO_2_ produced (VCO_2_), VE, breathing reserve/ventilatory reserve (VR), the ventilatory equivalent of O_2_ (VE/VO_2_), the ventilatory equivalent of CO_2_ (VE/VCO_2_), SpO_2_, AT, the heart rate (HR) and heart rate reserve (HRR). Extended parameters are valuable in special situations, such as congenital heart disease (CHD) and other chronic illnesses, where maximal exercise is impossible to include the oxygen uptake efficiency slope (OUES) [24]. Exercise testing can identify the physiological causes for exercise-related complaints and symptoms and assess (functional) the patient’s exercise capacity and exercise-limiting factors, including pathophysiological changes. CPET assesses the integrative exercise responses involving the pulmonary, cardiovascular, skeletal muscle, and cellular metabolic systems. These need to be adequately reflected by measuring individual organ system function. The CPET variables (independently and combined) offer useful prognostic information. Wassermann’s 9-plot uses several CPET variables as they interact [25]. Many reflect the ventilatory, cardiac, and metabolic inefficiencies that result from the extensive central and peripheral pathophysiological mechanisms in patients with cardiopulmonary diseases. The following main parameters are examined and interpreted at the end of the test.

### 4.1. Respiratory Equivalent Ratio (RER)

For an appropriate interpretation of CPET data, it is essential to determine whether the child performs a maximal or near-maximal effort. If the CPET is terminated prematurely, without maximally stressing the pulmonary, cardiovascular, and metabolic systems, this severely restricts the interpretation of the test. RER is the ratio of the VCO_2_ exhaled from the lungs over the VO_2_ absorbed from the lung in one minute. During CPET, if the RER is ≥1.1, it suggests a higher ability to perform in aerobic and anaerobic metabolism and represents a parameter close to maximum exhaustion [26].

### 4.2. Peak VO_2_ (PVO_2_)

Peak exercise capacity is defined as the maximum ability of the CV system to deliver O_2_ to exercising skeletal muscle and of the exercising muscle to extract O_2_ from the blood. PVO_2_ measures the peak exercise capacity in CPET, which is used as a synonym for maximum VO_2_ (VO_2_ max) throughout this review. In practice, PVO_2_ is the highest value reached during the maximal symptoms limited exercise test. It is a surrogate marker for the maximal cardiac output that an individual can achieve. PVO_2_ is better understood by analyzing the Fick principle, which estimates VO_2_ by the following equation: VO_2_ = stroke volume (SV) × heart rate (HR) × (CaO_2_–CvO_2_). In this equation, CaO_2_ is the arterial oxygen content, CvO_2_ is the venous oxygen content, and (CaO_2_–CvO_2_) is the arteriovenous (a−v) difference in O_2_. Thus, PVO_2_ is among the best-known and most frequently determined CPET variables. Conventionally, a PVO_2_ of >80% predicts or indicates adequate aerobic fitness [21]. While the New York Heart Association (NYHA) class is a clinical estimation of functional status, it is highly subjective. Hence, a more objective measure, i.e., PVO_2_, is helpful in better classifying the functional level in ambulatory heart failure (HF) patients. Weber’s classification characterizes the individual’s response to exercise as normal when the VO_2_ is >20 mL/min/kg (stage A), mildly to moderately impaired when the VO_2_ is ≤20 mL/min/kg (stage B), moderately to severely impaired when the VO_2_ is ≤16 mL/min/kg (stage C), and severely impaired when the VO_2_ is ≤10 mL/min/kg (stage D) [27]. It is essential to consider that the absolute PVO_2_ value is ∼10% higher on a treadmill than on a cycle ergometer [28]. A PVO_2_ < 14 mL/kg/min in ambulatory HF patients remains a cutoff for the optimal timing of evaluation for heart transplantation candidacy [29]. If the PVO_2_ values are ≥12 mL/min/kg or ≥14 mL/min/kg in those treated or not treated by β-blockers, respectively, they may not need an immediate heart transplant [30,31]. However, since the PVO_2_ is influenced by age, sex, and body weight, the absolute and the percent (%) of the predicted PVO_2_ values must be reported. The % predicted value might be a more reliable indicator of prognosis [32]. In children with dilated cardiomyopathy, the % predicted PVO_2_ values of <50% are associated with poor prognosis and are considered a criterion for heart transplant listing [33]. PVO_2_ also correlates with the NYHA functional class in adults with CHD [34]. However, the % PVO_2_ for each CHD is different, and one value does not fit all types of CHDs in children. Patients with single ventricle physiology (Fontan) have worse exercise capacity [35]. There are no extensive studies to establish the normal values for each CHD, but in general, PVO_2_ in children with CHD is significantly lower than in normal children [36,37]. Kempny et al. [38] have reported the reference values of PVO_2_ and other CPET variables for each CHD in adults and have correlated their data with those in the literature to guide those individuals’ recreational, sports, and professional activities [38].

### 4.3. Anaerobic Threshold (AT)

The AT, also known as the ventilatory anaerobic threshold, is defined as the VO_2_ at the onset of anaerobic metabolism, which is determined by using the rate of consumption of O_2_ and the elimination of CO_2_ during CPET. From this point, lactic acid builds up quickly in the blood due to the anaerobic metabolism of glucose and muscle glycogen. There are various methods for detecting VO_2_ at the AT, but the V-slope method, i.e., the point at which the VCO_2_ becomes higher, as compared with VO_2_, is due to the additional CO_2_ produced by lactic acid buffering. It is determined graphically when a sudden change in the slope of the regression line between VO_2_ and VCO_2_ occurs (the V-slope method) [39]. Additionally, the VE/VO_2_ increases at this transition compared to stable VE/VCO_2_ kinetics [40]. The exercise above the AT will create an acidic environment, shifting the O_2_ dissociation curve, thereby releasing O_2_ from hemoglobin more readily at a given degree of O_2_ content (the Bohr effect). In addition, the muscle temperature, CO_2_, and 2,3-diphosphoglycerate (2,3 DPG) will all contribute to a rightward shift in the O_2_ dissociation curve and benefit O_2_ unloading at the tissue level. The AT varies from athlete to athlete. With exercise training and regular high-intensity workouts, the AT in the muscles can be conditioned to a higher threshold of PVO_2_ and increased stamina [41]. A normal AT occurs at >40% of PVO_2_. During CPET, when the RER is <1.1, the VO_2_ at the AT is an essential submaximal marker of aerobic fitness rather than PVO_2_. It is also a critical variable that can be used for exercise training in children with CHD, where a heart rate target or PVO_2_ may not be achieved.

### 4.4. Ventilatory Equivalent for CO_2_ (VE/VCO_2_) and O_2_ (VE/VO_2_)

The VE/VCO_2_ responses to exercise also evaluate ventilatory efficiency, providing information about the effectiveness of the VE for a given metabolic rate. VE increases in response to CO_2_ production reflect an increased ventilatory drive [42]. The VE/VCO_2_ slope evaluation has the advantage over the determination of PVO_2_ because it can be obtained without maximal effort and is characterized by the time course of the gas exchange variables that reflect the adaptive capacity of cardiopulmonary function to the increasing work rates [43]. The normal VE/VCO_2_ value is <34. It is one of the independent prognostic markers, especially with submaximal effort, and has a critical prophetic role in HF, pulmonary artery hypertension, CHD, and lung diseases [44]. The prognostic importance of VE/VCO_2_ to clinicians and researchers is highlighted in the FUEL trial results. There is an improvement in VE/VCO_2_ rather than PVO_2_ after Udenafil in children with Fontan physiologies [45]. The VE/VCO_2_ slope is determined by the physiological dead space ventilation(V_D_)-tidal ventilation (V_T_) ratio (V_D_/V_T_) and the arterial CO_2_ partial pressure. A high VE/VCO_2_ slope suggests ventilation/perfusion mismatch, as seen in chronic HF, pulmonary vascular disease, and children with single ventricle CHD physiologies. Like VE/VCO_2_, the ventilatory equivalent of O_2_ (VE/VO_2_) increases because the VE disproportionately increases to eliminate excess CO_2_. The optimal cardiorespiratory point is the minimum VE/VO_2_ value (25–30). It is another submaximal variable that reflects the best integration between the respiratory and cardiovascular systems. VE/VO_2_ also reflects OUES and is a marker of ventilation–perfusion mismatch with the automatic calculation of OUES in most metabolic carts presently used. VE/VO_2_ is a less commonly used variable in CPET results analysis.

### 4.5. Oxygen (O_2_) Pulse

The amount of O_2_ consumed by the body from the blood of one systolic discharge of the heart is known as the O_2_ pulse. According to the Fick equation, the O_2_ pulse is the ratio of VO_2_ to HR per minute (expressed as mL O_2_/min). It reflects the amount of O_2_ extracted by exercising muscle per heartbeat. The O_2_ pulse estimates the left ventricle stroke volume changes during exercise, assuming that (a−v) O_2_ is maximal and no anemia or hypoxia is present [46]. The O_2_ pulse increases with exercise and gradually decreases to a plateau at peak exercise. A decreased O_2_ pulse during progressive exercise could indicate “pump” failure. A normal O_2_ pulse is >80% predicted [29]. A flat O_2_ pulse curve in adults was associated with effort-induced myocardial ischemia, probably reflecting myocardial dysfunction and low stroke volume [47]. Analysis of Washerman’s graphical representation of variables will enable us to learn the O_2_ pulse curve and increases in HR during a progressive increase in workload during exercise. In children with myocardial dysfunction, the O_2_ pulse curve flattens earlier than in normal children. The pattern of the O_2_ pulse helps determine the stroke volume and, thus, the contractility of the left ventricle.

### 4.6. Heart Rate (HR) and Heart Rate Reserve (HRR)

The first AHA guidelines for exercise testing in the pediatric age group emphasized HR and ECG changes with treadmills and cycle ergometers [48]. Variables that affect the HR during exercise depend upon the age and fitness level of the patients. In children, because of the small stroke volume, the heart rate is increased for a given rate of work and, thus, attains a higher maximal heart rate than adults. The maximal HR predicted is calculated from the formula: 220 − age in years and is commonly used as a basis for prescribing exercise programs. The maximally predicted HR is a criterion for maximal exertion and a clinical guide during diagnostic exercise testing [49]. A regression equation to predict the maximum HR in adults is 208 − 0.7 × age in years [50]. To a large extent, a predicted maximum HR is determined by age alone and is independent of gender and physical fitness status. Competitive athletes usually have lower HR increases and thus manage to sustain higher fractional PVO_2_ for a long time. Chronotropic incompetence is a failure to achieve 85% of the predicted heart rate during exercise. The heart rate is compared with nomograms for the stage of exercise and metabolic equivalent (MET) levels. The chronotropic index (the HR adjusted to the MET level) is common in children with Fontan physiologies. Analyzing the O_2_ pulse and HR response to exercise makes it easy to identify whether there is chronotropic incompetence or left ventricular “pump” dysfunction. A low chronotropic index is associated with mortality risks in patients with known CV diseases [51]. It is crucial to notice that the chronotropic index is low if the patient takes β-blockers. An unexpected increase in HR for MET is associated with physical deconditioning and/or anxiety and is helpful to differentiate from CV diseases. The heart rate reserve is the difference between the predicted HR for age and the actual HR achieved/predicted HR × 100 [50]. The heart rate reserve is usually normal in an average child and typically ≥15%, even with maximum exertion [50]. The heart rate reserve is decreased in physical deconditioning, whereas it remains normal with pulmonary limitations of exercise tolerance.

### 4.7. Ventilatory Reserve (VR)

Maximum voluntary ventilation (MVV) is the maximum resting volume of air that can be moved by voluntary effort in one minute [52]. The patient is instructed to breathe rapidly and deeply for 15 to 30 s, the ventilatory volumes are recorded, and the maximal volume achieved over 15 consecutive seconds is expressed in liters per minute. MVV can also be calculated using the formula FEV1 × 35 for females and FEV1 × 40 for males [50]. The VR is calculated as the ratio of maximal voluntary ventilation (MVV) at rest to maximal exercise minute ventilation (VE) [29]. Values of <30% suggest a ventilatory limitation and are helpful for differential diagnosis of dyspnea related to HF and those with chronic respiratory illnesses [53].

### 4.8. Oxygen Uptake Efficiency Slope (OUES)

The OUES is an important CPET variable for submaximal exercise, independent of effort. It is developed initially in children with CHD for medical clearance to engage in physical activities and medical indications for surgery [54]. It is defined by the linear relationship between VO_2_ and VE with VO_2_ on the *y*-axis and the log transformation of VE on the *x*-axis. This parameter is the physiological representation of the efficiency with which the O_2_ is extracted by the lungs and is used by the periphery [55,56]. OUES depends on age and the body surface area and should be expressed as OUES for the body surface area or body weight [55]. An OUES/body surface area of ≥1200 or an OUES of ≥35/body weight in kg correlates with the PVO_2_ reaching above 80% of the predicted values [57]. The OUES significantly decreases in children with CHD and pulmonary vascular disease and strongly correlates with PVO_2_ [58].

### 4.9. Oxygen Saturation (SpO_2_)

The accuracy of oximeters in measuring a change in SpO_2_ is ±2.5 to ±3.5 percent (95 percent confidence limits) [59]. A SpO_2_ of < 90% or a decrease of ≥4 percent from baseline is considered abnormal [60]. In the context of exercise testing, desaturation can occur most commonly in patients with diffusion limitations [61]. However, other pulmonary abnormalities, such as right-to-left shunts or ventilation–perfusion mismatching, may result in exercise-associated desaturation.

### 4.10. End-Tidal CO_2_ Partial Pressure (PETCO_2_)

The normal value ranges from 36 to 42 mmHg without significant lung diseases. PETCO_2_ reflects ventilation–perfusion within the pulmonary system and indirectly with cardiac function [25]. A high VE/VCO_2_ and low PETCO_2_ indicate a significant ventilation–perfusion mismatch and are characteristic of pulmonary vascular disease and chronic pulmonary thromboembolism [62]. Furthermore, it is worth mentioning that hyperventilation due to anxiety may also cause ventilation–perfusion mismatch. PETCO_2_ closely reflects PaCO_2_ in healthy individuals [63]. A PETCO_2_ of <36 is found in individuals with increased dead space ventilation, CHD with a right-to-left shunting, enhanced ventilatory drive, and/or tachypneic ventilatory pattern [64]. A PETCO_2_ of <36 is also associated with a blunted cardiac output response to exercise in patients with HF [65]. A markedly decreased PETCO_2_ suggests that the more likely cause of dyspnea is chronic pulmonary thromboembolism rather than idiopathic pulmonary hypertension [66].

### 4.11. Dead Space Ventilation (V_D_) and Tidal Ventilation (V_T_)

Tidal volume (V_T_) is the volume of gas inspired and expired during one respiratory cycle. Minute ventilation (VE) is the volume of gas exhaled per minute and the product between V_T_ and respiratory frequency. It is usually about 5–6 L/min but can rise in patients with a high production of CO_2_. V_D_/V_T_ further assesses the degree of mismatching of ventilation to perfusion during exercise if there is PaCO_2_ data available (the assumption of PaCO_2_ equals PETCO_2_ is valid only if there is no ventilation–perfusion defect). A low peak exercise PaCO_2_ (<35 mm Hg) and a high V_D_/V_T_ (>0.22) are both strong predictors of death in HF in adults [67]. However, children with the pulmonary disease have a significantly increased peak exercise V_D_/V_T_ (V_D_/V_T_ > 0.34) [68]. During rest, the V_D_/V_T_ values are usually higher than the exercise V_D_/V_T_ values_._ Exercise V_D_/V_T_ is an alternative measurement of ventilatory efficiency during exercise and possible right-to-left shunting.

### 4.12. Work Efficiency (VO_2_/WR)

Work is defined as the amount of exercise performed. It is only available for protocols using an ergometer. The units of work when a cycle ergometer is used are expressed as watts or joules. VO_2_/WR defines the total O_2_ cost of performing work and the aerobic contribution to exercise. A normal VO_2_/WR is 10–11 mL/min per watt [25]. A linear relationship exists between VO_2_ and WR during cycle ergometry testing, and a reduction in this ratio indicates CV diseases [69]. The loss of this linear relationship, combined with a reduction of VO_2_/WR < 5 mL/min per watt, despite increased exercise intensity during CPET, contributes to diagnosing myocardial ischemia in adults [70].

### 4.13. Circulatory and Ventilatory Power

Power is defined as the rate of performing work. The unit of power is the unit of work per unit of time. One joule per second is one watt. Circulatory power, a surrogate of peak cardiac power output, is the product of PVO_2_ (mL/kg/min) and peak systolic blood pressure (mmHg) [71]. Thus, applying Fick’s principle, it also represents the triple product of CO × C(a–v) O_2_ × systolic blood pressure. Incorporating an indirect measure of afterload (peak systolic pressure) seems to add predictive value for evaluating the results of functional status from CPET. Ventilatory power is a surrogate marker of ventilation and lung perfusion and is the ratio of the peak systolic BP and VE/VCO_2_ slope. The potential advantages of these two indices are that both are simple, noninvasive and synergistically combine singular indices related to cardiopulmonary functional status. Both variables add independent prognostic value for patients with HF, coronary artery disease [72], and pulmonary vascular disease [73].

### 4.14. VO_2_ Kinetics

VO_2_ kinetics describe the rate change in VO_2_ during exercise. It reflects the time required for the cardiopulmonary system to deliver an increased level of O_2_ and for the skeletal muscle to use that increased level of O_2_ needed for aerobic metabolism [74]. During incremental exercise protocols, VO_2_ kinetics can be estimated from the ratio of change in VO_2_ and change in WR and by the interval between the beginning of exercise and the linear increase in VO_2_ [74]. It has a significant added value during submaximal exercise. Patients with HF have prolonged VO_2_ kinetics, a more significant O_2_ deficit, a longer mean response time to a steady state, and a lower VE/VCO_2_ slope [75,76]. Heart failure interventions to improve endothelial function, peripheral circulation, and cardiac output have improved VO_2_ recovery kinetics. Finally, in cardiac patients with a moderately reduced exercise capacity, VO_2_ recovery kinetics may provide prognostic information [77]. However, prolonged VO_2_ kinetics are not specific to HF patients and may occur whenever O_2_ transport or O_2_ utilization by the exercising muscles is impaired, such as in anemia, hypoxia, peripheral artery disease, muscle deconditioning, and myopathies.

### 4.15. Periodic Breathing and Exercise Oscillatory Ventilation (EOV)

Kremser et al. [78] and Ribeiro et al. [79] described exercise-induced oscillatory ventilation (EOV) and periodic breathing in adult HF patients. This phenomenon is an extension of Cheyne-Stokes respiration seen during rest in patients with HF. During CPET, EOV is defined as the cyclic fluctuations in VE lasting more than 60% of the exercise duration, with an amplitude of more than 15% of the average cyclic changes at rest [80]. Other investigators identified EOV when at least two consecutive cycles of clear ventilatory oscillations were noted and when the mean difference between the peak and the nadir of oscillating VE was more than 30% of the mean value of VE [81]. EOV has an independent prognostic value in HF. Therefore, the CPET report should include the frequency, amplitude, and duration of EOV. It is also essential to underscore the presence of EOV during exercise, which may cause variable artifacts in determining PVO_2_, RER, and AT.

## 5. Interpretation of CPET

**Figure 1:**
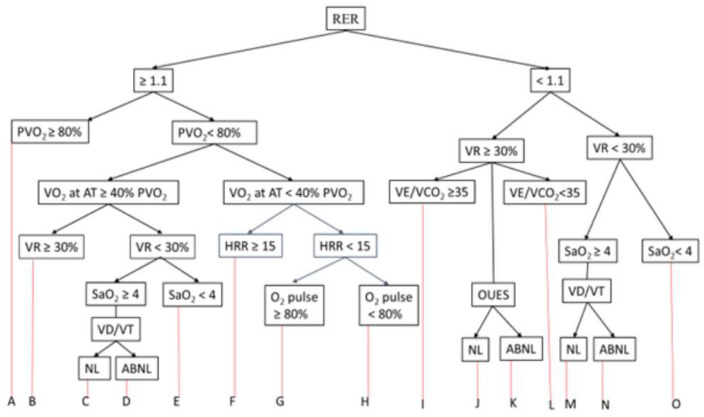
RER: respiratory exchange ratio; PVO_2_: peak oxygen consumption; VR: ventilatory reserve; AT: anaerobic threshold; VE/VCO_2_: ventilatory equivalent for carbon dioxide; HRR: heart rate reserve; SaO_2_: oxygen saturation; OUES: oxygen uptake efficiency slope; VD/VT: dead space ventilation/tidal ventilation; NL: normal; ABNL: abnormal.

An integrated approach uses multiple variables to aid in more precise risk stratification and decision processing. It is also noteworthy that many variables are physiologically related, e.g., PVO_2_ and circulatory power, VE/VCO_2_, and PETCO_2_. Despite these limitations, I propose an algorithm (Figure 1) incorporating the commonly obtained CPET variables to differentiate the cause of exercise intolerance. Table 3 describes the interpretation from A to O that resulted from the algorithm. However, more studies are needed to validate the algorithm proposed in this paper.

## 6. Conclusions

CPET is the most comprehensive test that integrates multiple data and allows for assessing the cardiovascular, respiratory, muscular, and metabolic systems during exertion. It is considered the gold standard for cardiorespiratory functional assessment. The clinical application of CPET continues to evolve, and protocols should be adapted to each specific patient to obtain the most reliable and helpful diagnostic data. In addition to aiding in a more careful exercise prescription for patients with CV and/or respiratory diseases, it can provide a prognostic assessment of patients with heart or lung diseases, as well as in the preoperative period.

## Figures and Tables

**Table 1:** Comparison of Cardiopulmonary variables at rest vs. exercise.

Physiological Variables	Rest	Exercise
Heart Rate/min	75	Increase 2 to 2.5 times
Cardiac cycle in seconds	0.8	0.35
LV end-diastolic volume (mL)	60–145	150–180
Systolic blood pressure (mean) mmHg	120	Increases by 30–40%
Cardiac Output (L/min)	3–5	Increases by 3–5 times
Arteriovenous oxygen difference (mL/dL)	3.5–5	7–10
Oxygen Uptake (mL/kg/min)	3.5	Increases by 3–5 times
Myocardial oxygen usage		Increases by 3–5 times
Breathing rate/min	12–16	40–50
Tidal volume (mL)	500	2300 to 3000
Minute ventilation (L/min)	5–6	100
Pulmonary capillary blood transit time (s)	0.75	Decrease (0.38)
Alveolar-arterial oxygen difference (mmHg)	10	20–30

**Table 2:** CPET variables.

Maximum voluntary ventilation (MVV)
Heart rate (HR)
Blood pressure (BP)
Respiratory exchange ratio (RER)
Peak oxygen consumption (Peak VO_2_)
Anaerobic threshold (AT)
Ventilatory equivalents: VE/VO_2_, VE/VCO_2_ Slopes
Oxygen pulse
Heart rate reserve (HRR)
Ventilatory reserve (VR)
Oxygen uptake efficiency slope (OUES) Oxygen saturation (SpO_2_)
End-tidal CO_2_ partial pressure (PETCO_2_)
Dead space ventilation/Tidal volume ventilation (V_D_/V_T_)
Work efficiency (VO_2_/WR)
Circulatory Power
Ventilatory power
Oxygen kinetics
Exercise oscillatory ventilation (EOV)

**Table 3:** Interpretation using the algorithm (Figure 1).

A. No pulmonary or circulatory limitations (Normal CPET)
B. Mild circulatory limitation
C. Moderate gas exchange abnormality
D. Moderate mechanical ventilation abnormality
E. Mild gas exchange abnormality (exercise-induced bronchospasm)
F. Moderate circulatory impairment
G. Physical deconditioning
H. Cardiac limitation due to “pump” dysfunction
I. Moderate ventilation-perfusion abnormality
J. Poor effort
K. Modretae circulatory limitation
L. Moderate gas exchange abnormality
M. Severe mechanical ventilation abnormality
N. Mixed cardiac and pulmonary limitation.
O. Physical deconditioning

## Data Availability

Not applicable.
